# Targeting Nuclear Hormone Receptors: PPAR**α** Agonists as Potential Disease-Modifying Drugs for Rheumatoid Arthritis

**DOI:** 10.1155/2011/937843

**Published:** 2011-06-21

**Authors:** Ivan V. Shirinsky, Valery S. Shirinsky

**Affiliations:** Laboratory of Clinical Immunopharmacology, Scientific Research Institute of Clinical Immunology, RAMS, 14 Yadrintsevskaya Street, Novosibirsk 630099, Russia

## Abstract

In recent years, peroxisome proliferator-activated receptors (PPARs) have received growing interest due to the broad spectrum of their biological activities. PPAR**α**, an isoform of PPAR, plays an important role in lipid homeostasis and inflammation, which makes it a potential target for the treatment of chronic inflammatory disorders, including RA. This paper reviews studies on the properties of PPAR**α** agonists which may be pertinent to the treatment of RA. These properties include effects on lipid metabolism, inflammation, and angiogenesis, as well as interference with glucocorticoid effects, and a potential role in gender dimorphism of autoimmune disorders. However, current clinical experience with this class of drugs in RA is limited. New studies are needed to elucidate whether PPAR**α** agonism may be an effective treatment strategy for RA patients.

## 1. Introduction

The nuclear hormone receptor superfamily is a large group of related receptors which are able to bind a broad-ranging array of ligands. The peculiarity of nuclear receptors is that upon activation, they act as transcription factors binding to a specific DNA sequence resulting in changes in gene expression. The nuclear receptor superfamily is divided into six subfamilies and 26 groups of receptors. Subfamily 1 is represented by peroxisome proliferator-activated receptors (PPARs) (Nuclear Receptors Nomenclature Committee, 1999) [1], which play a major role in lipid metabolism, glucose homeostasis, and inflammatory processes. Three isotypes of PPAR have been described: (1) PPAR*α*, also known as nuclear receptor subfamily 1, group C, member 1 (NR1C1), (2) PPAR*β*/*δ* (NR1C2), and (3) PPAR*γ* (NR1C3). These isotypes have different tissue distribution, functions, and ligand specificity. In particular, PPAR*α* is highly expressed in the liver, heart, brown adipose tissue, skeletal muscle, and kidney. Its expression has also been proven on dendritic cells, macrophages, and B and T cells [2]. There are both natural and synthetic ligands of PPAR*α*. Endogenous ligands are mainly unsaturated or polyunsaturated fatty acids and eicosanoids and need to be at micromolar concentrations to achieve PPAR activation [3], except 1-palmitoyl-2-oleoyl-sn-glycerol-3-phosphocholine (16 : 0/18 : 1 GPC) which has nanomolar affinity [4]. Synthetic agonists of PPAR*α* are hypolipidemic drugs (fenofibrate, gemfibrozil, clofibrate, nafenopin, methyl clofenapate, tibric acid, and Wy-14,643) which act at the nanomolar range. PPAR*α* has been proposed as a key lipid metabolism modulator and regulator of inflammation [2]. Therefore, these properties of PPAR*α* make it a possible target for therapy in rheumatoid arthritis (RA), which is characterized by accelerated atherosclerosis and impaired lipid profile [5]. This paper will summarize the data on PPAR*α* biological functions with implications to the treatment of autoimmune disorders as well as the current clinical experience with PPAR*α* agonists in RA. 

## 2. PPAR*α* and Lipid Metabolism

**Figure 1:**
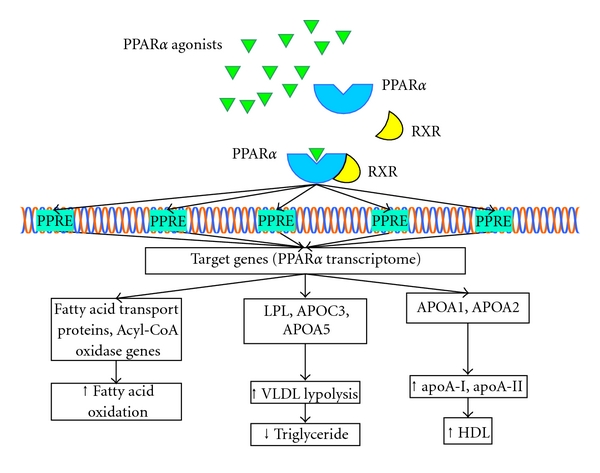
PPAR*α* and lipid metabolism. PPAR*α* forms heterodimers with RXR. The heterodimers bind to PPREs which leads to enhanced expression of many genes involved in lipid metabolism. The main resulting changes are increased fatty acid oxidation, decreased triglyceride concentration, and increased levels of HDL. RXR: retinoid X receptor, PPRE: peroxisome proliferator response elements, and HDL: high density lipoprotein.

PPAR*α* induces gene transcription after forming heterodimers with the 9-*cis* retinoic X receptor (RXR). Then these heterodimers bind to specific DNA sequences called Peroxisome Proliferator Response Elements (PPREs) in the promoter regions of multiple target genes forming the so-called PPAR*α* transcriptome (Figure 1) [6]. 

In the liver, activation of PPAR*α* promotes fatty acid oxidation, ketone bodies synthesis, and glucose sparing via the induction of various protein synthesis such as fatty acid transport proteins and acyl-CoA oxidase [2]. 

In terms of lipoprotein metabolism, PPAR*α* activation results in changes in transcription of multiple genes including LPL, APOC3, PCKK9, ANGPTL3, APOA1, APOA2, and APOA5 [7]. A well-known effect of fibrates is a reduction in plasma triglyceride levels. This is thought to be a result of enhanced lypolysis of very low density lipoprotein (VLDL) triglyceride induced by changes in LPL, APOC3, and APOA5 transcription. APOA1, APOA2 transcription changes result in enhanced apoA-I and apoA-II production leading to increased high density lipoprotein cholesterol (HDL-c) concentrations [7]. 

The lipid-modulating properties of fibrates suggest that they may improve impaired lipid profile observed in RA patients (Table 1). Thus, although triglycerides are less strongly associated with cardiovascular risk in RA patients than in people without RA [8], their reduction induced by fibrate treatment may be of benefit. Moreover, in one study it has been shown that, under fibrate treatment, only triglycerides were independent predictors of CHD [9]. 

Another important lipid target of fibrates is HDL-c whose concentrations are decreased in RA and have been linked to excess cardiovascular events in some studies [10]. 

Apart from their beneficial action, fibrates may have some undesirable metabolic effects, particularly increased homocysteine levels [11]. Homocysteine reduces apoA-I synthesis in the liver leading to decreased plasma apoA-I levels [12]. It has been shown that higher homocysteine levels correlate with smaller increases in HDL and apoA-I after fenofibrate treatment [13]. Fenofibrate effects on oxidized low density lipoprotein (oxLDL), which is elevated in RA and probably linked with cardiovascular morbidity [14], are also diminished by high levels of homocysteine [15]. It should be noted that homocysteine itself is an independent risk factor of cardiovascular disease in RA [16], although, to date homocysteine-lowering treatment has not been proven to be effective in reducing cardiovascular outcomes, possibly due to not taking into account baseline homocysteine concentrations [17]. As the majority of patients with RA are now taking folate as supplementation to methotrexate treatment, it may lead to serum homocysteine reduction and thus improve lipid-modulating effects of fenofibrate, enhancing its action on HDL, apoA-I, and oxLDL. 

## 3. Anti-Inflammatory Action of PPAR*α*

A number of in-vitro studies exploiting different experimental models have investigated effects of PPAR*α* agonists on inflammation markers. It has been found that PPAR*α* agonists inhibited inducible nitric-oxide synthase activity in murine macrophages [21], and VCAM-1 expression in endothelial cells [22]. In human aortic smooth muscle cells (SMC), PPAR*α* agonists reduced IL-1 induced production of IL-6, prostaglandin, and expression of COX-2 [23, 24]. In addition, PPAR*α* ligands induced apoptosis of human monocyte-derived macrophages activated by TNF-*α* or IFN-*γ* [25].

The first evidence of the in-vivo anti-inflammatory action of PPAR*α* agonists in humans came from the studies performed on patients with hyperlipidemia and metabolic syndrome. Thus, fenofibrate treatment decreased plasma concentrations of IL-6, fibrinogen, and C-reactive protein [24] in hyperlipidemic patients. In another study performed on hyperlipoproteinemia IIb and atherosclerosis patients, micronized fenofibrate reduced serum TNF-*α* and IFN-*γ* concentrations [26]. These results have been confirmed by a small randomized placebo-controlled study in patients with metabolic syndrome, showing decreases in high-sensitivity C-reactive protein and IL-6 levels following fenofibrate therapy. These fenofibrate effects were independent of its effects on lipid and glucose metabolism [27]. 

**Figure 2:**
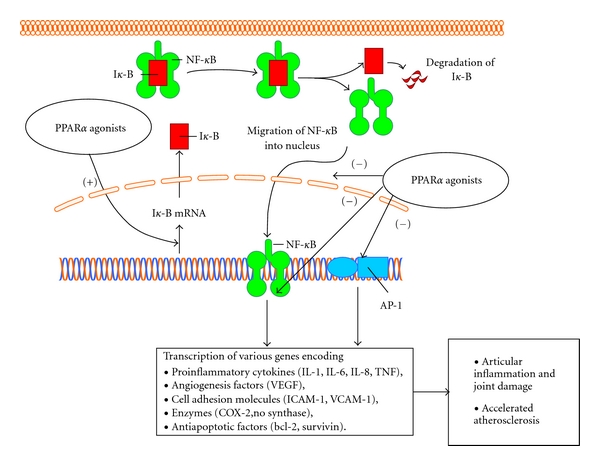
A hypothetical model for PPAR*α*-induced anti-inflammatory effects in rheumatoid arthritis. PPAR*α* might suppress transcriptional activity of NF*κ*B by several mechanisms: directly, by inducing I*κ*B transcription or by inhibition of NF*κ*B migration into nucleus. Another transcription factor AP-1 is also suppressed by PPAR*α*. Down regulation of NF*κ*B and AP-1 results in reduced synthesis of various mediators involved in joint inflammation and damage as well as in atherosclerotic plaque formation. NF*κ*B: nuclear factor *κ*B, I*κ*B: inhibitor of *κ*B, AP-1: activator protein 1.

Several studies have sought to characterize the molecular mechanisms implicated in the downmodulation of inflammatory mediators by PPAR*α* activation. As a result, it has been demonstrated that PPAR*α* exerts its effects on proinflammatory cytokine gene expression by antagonizing the AP-1 and nuclear factor *κ*B (NF-*κ*B) transcriptional activities in human aortic SMC [24, 28]. An additional molecular mechanism of PPAR*α* agonists' anti-inflammatory action is induction of the expression of the NF-*κ*B inhibitory protein I*κ*B*α* found in SMC as well as in primary human hepatocytes (Figure 2) [29].

The biological role of PPAR*α*-induced anti-inflammatory effects seems to be the control of inflammatory response duration. This control is probably mediated by endogenous PPAR*α* ligand leukotriene B_4_ (LTB4), which is a powerful chemotactic inflammatory eicosanoid. PPAR*α* activation leads to transcription of genes of the *β*- and *ω*-oxidation pathways that neutralize and degrade LTB4 itself, thus regulating inflammation by a negative feedback loop [30]. 

The experimental and clinical studies relevant to atherosclerosis and dyslipidemia were followed by the work of Okamoto et al. [31] who assessed the anti-inflammatory effects of PPAR*α* activation in rheumatoid synovial fibroblasts (RSF) cultures and in a rodent model of inflammatory arthritis. Fenofibrate reduced IL-1*β*-stimulated production of IL-6, IL-8, and GM-CSF as well as nuclear translocation of NF-kB in RSF. The therapeutic use of fenofibrate leads to clinical improvement and inhibited mononuclear cell infiltration and reduced pannus formation in the synovial tissue of rats with adjuvant-induced arthritis. Moreover, fenofibrate inhibited osteoclast formation from human peripheral blood mononuclear cells in-vitro. 

Several other studies evaluated the effects of PPAR*α* agonists on cytokine production in different experimental settings. Thus, fenofibrate repressed interleukin-17 and interferon-gamma expression and decreased colonic lymphocyte infiltration in a colitis model in interleukin-10-deficient mice [32]. Fenofibrate treatment also resulted in clinical improvement and enhanced cardiac expression of IL-10 mRNA in a rat model of experimental autoimmune myocarditis [33].

Taken together, these data give a rationale for PPAR*α* agonists to be evaluated both as modulators of the inflammatory response and as a disease-modifying class of drug in RA. 

## 4. PPAR*α* Interference with Glucocorticoid Effects

It is known that PPAR*α* is activated by glucocorticoids (GC) during fasting or stress [34]. Recently, it has become apparent that PPAR*α* may itself modulate multiple biological effects of GC. 

Genomic mechanisms of GC action are mediated by binding of GC to cytosolic GC receptors (cGCR). Then GC/cGCR complex is translocated into the nucleus to consensus palindromic DNA sites, which are called GC responsive elements (GRE) [35]. Genes regulated by GRE encode proteins involved in glucose, fat, and protein metabolism. Alternatively, activated cGCR monomers can also influence gene expression by interfering with the activity of transcription factors NF-*κ*B and AP-1, which play a key role in inflammatory mediator synthesis. There is a broad consensus that GCs exert their anti-inflammatory effects via transrepression of NF-*κ*B and AP-1 whereas detrimental side effects originate from the transactivation capacities of GR mediated by GRE binding [35].

There have been several studies evaluating interactions between the effects of GC and PPAR*α* activation. Riccardi et al. studied anti-inflammatory effects of dexamethasone on experimental inflammatory bowel disease in PPAR*α* knockout mice in comparison with wild type mice. The authors found that dexamethasone was less effective in PPAR*α* null mice as evaluated by inhibition of proinflammatory cytokine production, cell migration, oxidative stress, apoptosis, and colon injury. These findings indicate that PPAR*α* agonism may contribute to the anti-inflammatory action of GC [36]. 

**Figure 3:**
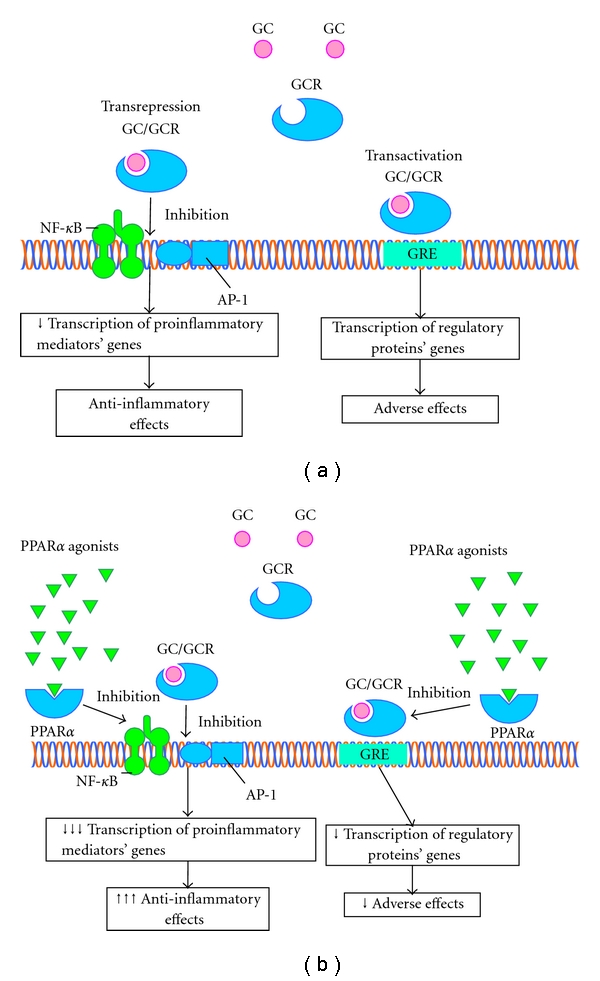
(a) a hypothetical mechanisms of PPAR*α* interference with glucocorticoid effects. GC/GCR complex binds to specific DNA sites called GRE which results in increased expression of many genes encoding proteins involved in fat, glucose, and protein metabolism. Adverse effects of GC are thought to stem from GRE binding. GC/GCR also down regulates transcription factors NF*κ*B and AP-1 thus suppressing synthesis of inflammatory mediators. (b) PPAR*α* further inhibits NF*κ*B and AP-1 thus enhancing GC anti-inflammatory action. PPAR*α* inhibition of GC/GCR-mediated GRE activation leads to attenuation of GC-induced adverse events. GC: glucocorticoid, GCR: glucocorticoid receptor, GRE: glucocorticoid response element, NF*κ*B: nuclear factor *κ*B, I*κ*B: inhibitor of *κ*B, and AP-1: activator protein 1.

To elucidate molecular mechanisms of PPAR*α* and GC synergism, Bougarne et al performed a study evaluating a functional cross-talk between PPAR*α*- and GCR-mediated signaling pathways. As was expected, simultaneous activation of PPAR*α* and GCR enhanced transrepression of NF-*κ*B-driven genes and additively decreased proinflammatory cytokine production. On the other hand, PPAR*α* activation inhibited the expression of classical GRE-driven genes, thus acting as a potential antagonist to GC with respect to their effects on glucose, fat, and protein metabolism [37]. So it can be hypothesized that PPAR*α* agonists attenuate GC side effects while enhancing their anti-inflammatory activity via transrepression of NF-*κ*B (Figure 3). 

## 5. PPAR*α* and Angiogenesis

Angiogenesis, or formation of new capillaries from preexisting vessels, is a characteristic feature of inflamed synovium in RA and develops at the earliest stage of the disease process. Angiogenesis is essential for the formation of the inflammatory pannus, and without angiogenesis, leukocyte migration could not occur [38]. 

The role of PPAR*α* in angiogenesis is controversial. In one study, fenofibrate was shown to inhibit endothelial cell proliferation induced by angiogenic factors, endothelial cell migration in a healing wound model, capillary tube formation in-vitro, and angiogenesis in-vivo [39]. Other research has demonstrated antiangiogenic effects of fibrates leading to suppressed tumor growth [40]. In contrast, fenofibrate enhanced neovascularization in a murine hind-limb ischemia model [41] and in a murine corneal model of angiogenesis [42]. 

Modulatory effects of PPAR*α* on angiogenesis seem to be mediated by changes in the expression of different pro-angiogenic modulators, such as VEGF, fibroblast growth factors (FGF), thrombospondin, and endostatin [43]. 

In contrast with angiogenesis, vasculogenesis, which is de novo capillary formation from endothelial precursor cells (EPCs), is impaired in RA. Deteriorated function of EPC may lead to changes in vasculogenesis resulting in accelerated atherosclerosis and vascular disease [44]. 

Using a PPAR*α*−/− mouse model, Benameur et al. have demonstrated that EPC differentiation induced by microparticles (small vesicles released from the plasma membrane of stimulated or apoptotic cells) is dependent on PPAR*α* and mediated by the NF-*κ*B pathway [45]. On the basis of this study, it may be speculated that PPAR*α* agonists improve vasculogenesis via stimulation of EPC. 

Thus, the effects of PPAR*α* on angiogenesis and vasculogenesis seem to be multidirectional and probably depend on the local cytokine and growth factor balance as well as the disease model studied. 

## 6. PPAR*α*, Sexual Dimorphism, and Autoimmune Diseases

Apart from its importance in energy metabolism and inflammation, PPAR*α* has been shown to play a role in sexual dimorphism partly due to an ability to regulate female-specific gene expression in the liver [46]. Dunn et al. have further demonstrated that this aspect of PPAR*α* biology might be relevant in the context of autoimmune disease pathogenesis. In their study, they found that PPAR*α* was more abundant in male as compared with female CD4 (+) cells and its expression was sensitive to androgen levels. Genetic ablation of PPAR*α* resulted in higher production of IFN-*γ* and TNF-*α*, and lower production of T helper (Th)2 cytokines due to upregulation of NF-*κ*B and c-jun activity in male T lymphocytes. Moreover, male, but not female, PPAR*α* (−/−) mice developed more severe experimental autoimmune encephalomyelitis. The authors' conclusion is that males are less prone to develop Th1-mediated autoimmunity because they have higher T-cell expression of PPAR*α* [47]. These findings allow one to hypothesize that PPAR*α* may be important in gender dimorphism in human autoimmune disorders including RA. 

## 7. Clinical Use of PPAR*α* Agonists in RA

To date, the clinical experience with PPAR*α* agonists in RA is limited. First, a case report on a female patient with refractory RA taking fenofibrate showed long-lasting improvement of her symptoms [48]. Goto reported a randomized study of 44 RA patients comparing fenofibrate and statins. Fenofibrate, but not statins, significantly decreased serum levels of total cholesterol, low density lipoprotein cholesterol (LDL-C), and triglycerides. In comparison with statins, fenofibrate significantly reduced prednisolone use. Unfortunately, the author has described neither changes in composite disease activity measures nor clinical response rates after fenofibrate therapy [49]. 

## 8. Conclusion

There is a substantial body of data suggesting that PPAR*α* may be of benefit in patients with RA due to their anti-inflammatory and lipid-modulating properties. Proof-of-concept studies are needed to assess efficacy and safety of PPAR*α* agonists in autoimmune diseases including RA and to address the issues arising from our current understating of PPAR*α* agonists pharmacology (Table 2). 

## Figures and Tables

**Table 1:** Some metabolic effects of PPAR*α* agonists with their relevance to RA.

Parameter	Effect of PPAR*α* agonists	Relevant changes in RA
TG	↓ [18]	TG levels are weakly related to ischaemic stroke [8]
HDL	↑ [19], higher effect correlates with lower serum homocysteine concentrations [13]	HDL-c levels are decreased [10]
Total cholesterol	↓ [18]	No changes in total cholesterol revealed in a recent meta-analysis [10]
oxLDL	No effect due to increase in homocysteine, folic acid coadministration may potentiate fenofibrate antioxidative capacity directed on oxLDL [15]	Increase of oxLDL concentrations [14]
Homocysteine	↑ [20]	Homocysteine concentrations are increased; however, this may be corrected by folic acid intake [16]

TG: triglyceride, HDL: high density lipoprotein, oxLDL: oxidized low density lipoprotein, ↑: increase, ↓: decrease.

**Table 2:** Future research agenda.

(1) Anti-inflammatory, disease-modifying, and antiatherogenic properties of PPAR*α* agonists in RA patients have to be tested in a randomized, placebo-controlled fashion.	
(2) PPAR*α* agonists' ability to enhance the anti-inflammatory action of glucocorticoids and reduce their side effects in inflammatory rheumatic disorders should be assessed.	

(3) The hypolipidemic and antiatherogenic effects of PPAR*α* agonists need to be compared between subgroups of RA patients taking or not taking supplemental folic acid.	
